# Hyperopic reserve as a predictor of myopia incidence in schoolchildren aged 6–12 years: a 24-month prospective cohort study

**DOI:** 10.3389/fpubh.2025.1660168

**Published:** 2025-09-29

**Authors:** Wei Dong, Kunlin Fu, Ye Zhang, Yangbing Li, Chengfei Liu, Zihao Wang, Yanying Li, Boyang Song, Zhaojiang Du

**Affiliations:** ^1^Department of Ophthalmology, Xi’an Central Hospital, Xi’an, China; ^2^Medical College of Yan’an University, Yan’an, China; ^3^Department of Ophthalmology, Maternal and Child Health Care Hospital of Dingbian County, Yulin, China; ^4^Department of Organ Donation, The First Affiliated Hospital of Xi’an Jiaotong University, Xi’an, China

**Keywords:** myopia, hyperopic reserve, schoolchildren, risk stratification, prospective cohort study, northwest China

## Abstract

**Purpose:**

To investigate the age-specific prevalence of hyperopic reserve deficiency and its predictive value for incident myopia among 6-12-year-old schoolchildren in Xi’an, China.

**Methods:**

This prospective cohort study enrolled 15,046 students from 30 primary schools. Diopter and visual acuity were assessed at baseline (2021) and follow-up (2023). Hyperopic reserve thresholds followed Chinese national guidelines (SE: <+1.00D for ages 6–8, <+0.50D for age 10). Multivariable logistic regression identified risk factors for myopia progression.

**Results:**

Myopia prevalence increased significantly with age (10.25% at 6 years to 49.77% at 12 years; *p* < 0.001). Females exhibited higher overall myopia rates (26.38% vs. 21.26%; *p* < 0.001). Hyperopic reserve deficiency peaked at age 8 (91.62%) and declined thereafter. Children with baseline reserve −0.5D to 0D demonstrated a 40.27% myopia conversion rate, compared to 3.33% in those with > + 2.00D (*p* < 0.001). Key risk factors included parental myopia (OR = 1.60, 95% CI:1.42–1.80, *p* < 0.001 for maternal, OR = 1.35, 95% CI:1.20–1.54, *p* < 0.001 for paternal), prolonged study time (OR = 1.49 95% CI: 1.33–1.68, *p* < 0.001), prolonged screen time (OR = 1.17, 95% CI:1.04–1.30, *p* = 0.007), and high sugar intake (OR = 1.46, 95% CI:1.30–1.64, *p* < 0.001), while outdoor activity (OR = 0.47, 95% CI:0.40–0.55, *p* < 0.001), adequate sleep (OR = 0.84, 95% CI:0.75–0.95, *p* = 0.004) and good learning posture were protective(OR = 0.63, 95% CI:0.56–0.71, *p*<0.001).

**Conclusion:**

Hyperopic reserve −0.5D to 0D identifies high-risk subgroups for myopia development, particularly among females. Preventive interventions targeting reserve preservation should commence by age 6, integrating school-based strategies and personalized clinical protocols.

## 1 Introduction

The global myopia pandemic disproportionately affects East Asia, with prevalence exceeding 80% among urban adolescents (1). In China, myopia-related complications now account for 12.8% of pediatric healthcare expenditures, driven by rapid urbanization and educational pressures (2). Ophthalmic biometric features such as axial length (AL), corneal curvature, and lens power are among the most important factors affecting the refractive state of the eye. During infancy and early childhood, most children have a physiological hyperopia of approximately +2.00 diopters (D), which is defined as hyperopic reserve (3). Balancing changes in AL and ocular refractive components, including the cornea and lens, leads to emmetropia (4). As children age, the refractive power of the eye develops, and the hyperopic reserve gradually decreases (5). It is widely believed that changes in myopic refractive power in schoolchildren are caused by axial elongation (6). When the rate of axial elongation exceeds the changes in the optical power of the cornea and lens, there is a tendency for myopia to develop. For young children, early reduction in hyperopic reserve may be a risk factor for future myopia development. It has been established that hyperopic reserve—the refractive buffer against premature ocular elongation—serves as a critical biomarker for myopia prediction, yet regional disparities in reserve dynamics remain underexplored (7).

It has been reported that in the Chinese population, the myopic cutoff point for first-grade students is +0.31 D (8). Meanwhile, the onset age of myopia has shifted toward younger populationsin recent years. For instance, among Chinese children, the average onset age decreased from 12 years old in 2010 to 7 years old in 2019 (9). Studies have also shown that myopia develops most rapidly between the ages of 6 and 7, and tends to slow down after the age of 11 (10). However, current guidelines lack evidence-based thresholds for initiating interventions during the critical 6-8-year window (11). In a study conducted in Jiading District, Shanghai, the results of a 1-year cohort indicated that, besides the duration of outdoor and close work, behaviors related to close work are also associated with the occurrence of myopia in schoolchildren (12). Another study followed up on primary school students for 4 years, from 2010 to 2014, and measured their non-cycloplegic refractive parameters in Baoshan District, another suburb of Shanghai, which showed that Incidence and progression of myopia is relatively high in schoolchildren in Shanghai compared with children of Western countries, East Asia and other parts of China (11). While coastal Chinese cities like Shanghai dominate existing research, inland regions like Xi’an (latitude 34°16’N) face unique challenges, including lower urbanization rates (74.6% vs. Shanghai’s 88.3%) and limited access to preventive care (13). This 24-month cohort study addresses three gaps: (1) characterizing hyperopic reserve depletion patterns in Northwest China, (2) quantifying sex-specific myopia conversion rates, and (3) establishing actionable reserve thresholds for precision prevention. This study systematically analyzes and discusses the accuracy, cutoff values, and feasibility of hyperopic Reserve as a predictor in screening myopia, providing a reference for the practice of myopia screening. The purpose of myopia screening is early identification and early intervention, which means that how to improve the referral and intervention rates after screening plays a crucial role in myopia prevention and control.

## 2 Method

### 2.1 Study design and participants

**Figure 1:**
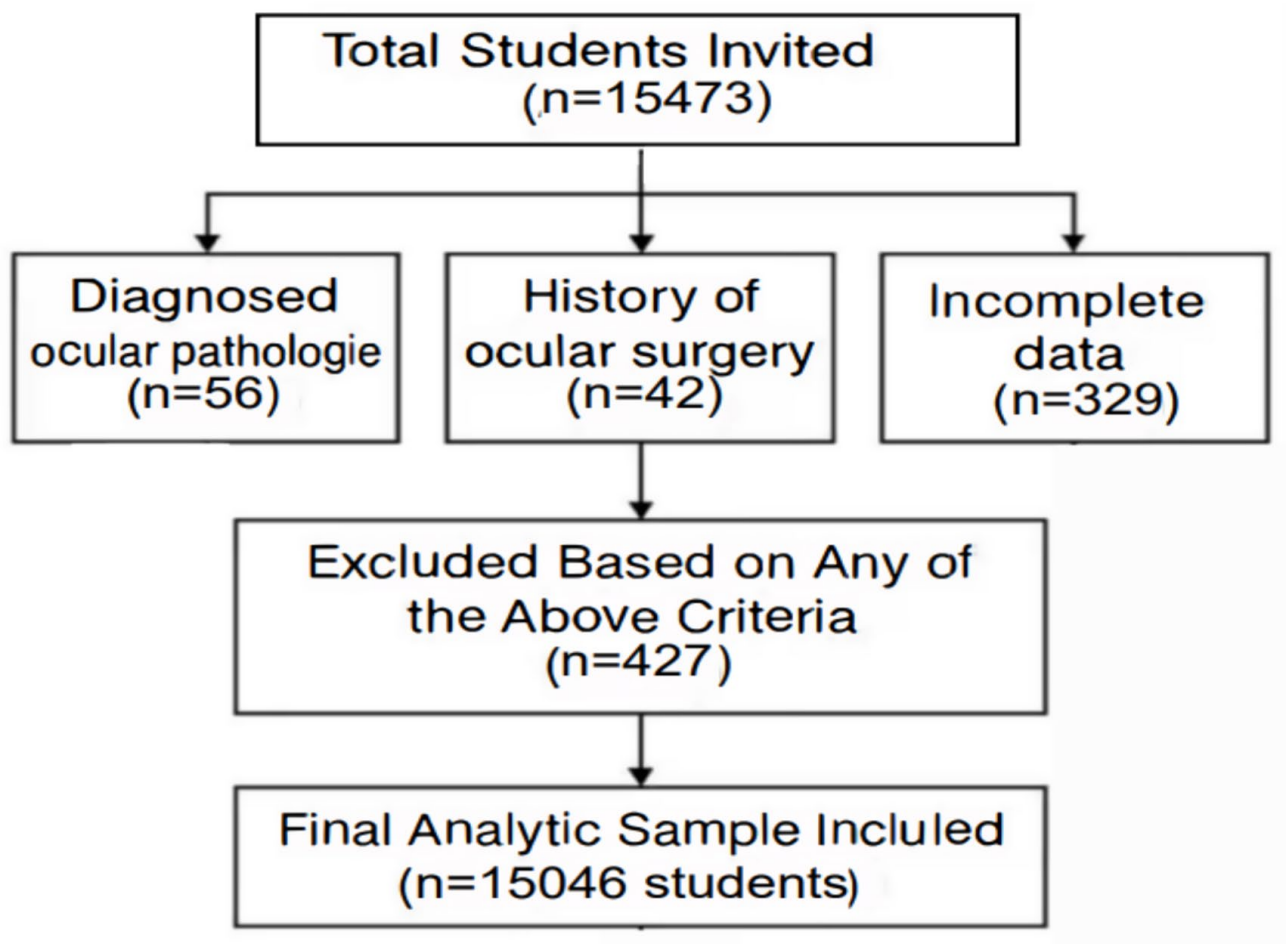
A flowchart showing the students affected by the various exclusion criteria.

The sample size was based on an estimated one-year incidence rate of myopia of 25.7%, with a 95% confidence interval (CI) limit of ±5% (*α* = 0.05, *d* = 0.1p) (12). Cluster randomization based on probability and size proportion was used. Schools were stratified based on prevalence of myopia. School-based cluster randomization was chosen in this study. Based on the stratified cluster sampling approach, 15,473 students aged 6–12 years from 30 primary schools in Xi’an (September–November 2021) were recruited in this study. Exclusion criteria included ocular pathology, or incomplete follow-up (*n* = 427). The final cohort comprised 15,046 participants (97.2% retention). This study adhered to the tenets of the Declaration of Helsinki and was conducted in accordance with institutional and national ethical standards. The Ethics Committee of the Xi’an Central Hospital approved this study. Moreover, the procedure was explained to the participants and their parents and then informed written consent was obtained from parents or legal guardians before entering the study. The follow-ups were carried out every 6 month during this study. During each followup section, visual acuity (VA), non-cycloplegic refractive power (NCR), axial length (AL), and corneal radius were tested for all eligible participants. Participants also underwent tonometer and slit lamp tests to help diagnose eye diseases such as glaucoma and to identify contraindications to cycloplegia. All tests were conducted by designated personnel who underwent training and qualification review before the commencement of this study. The students were grouped bases on age. A flowchart showing the students affected by the various exclusion criteria (Figure 1).

### 2.2 Measurements

#### 2.2.1 Refractive assessment

In the case of non-ciliary muscle paralysis, a fully automatic computer optometer (Topcon RM-800) was used to measure the spherical equivalent by a professional optometrist. Three consecutive measurements (SD < 0.50D) were averaged.

#### 2.2.2 Visual acuity

Before cycloplegia, the visual acuity measurement is examined by a trained ophthalmologist or technician Using a mounted and illuminated E chart of the Early Treatment Diabetic Retinopathy study (ETDRS) charts (LCD backlit lamp, 400cd/m2, WH0701, Guangzhou Xieyi Weishikang) at 5 meters using ambient room lighting.

#### 2.2.3 Questionnaire

Parental myopia, daily activities (learning, screen time, outdoor exposure,sleep time), dietary habits and learning posture were recorded. This questionair has been validated in Chinese school children previously (12).

### 2.3 Definitions

Screening myopia: visual acuity < 1.0 in the unaided vision and SE ≤ −0.50D as measured by a computer optometer. Students wearing orthokeratology lenses were included in the myopia group. Hyperopia reserve is considered as a physiological hyperopic refractive status that precedes emmetropia and myopia. It has been established that hyperopic reserve—the refractive buffer against premature ocular elongation—serves as a critical biomarker for myopia prediction, yet regional disparities in reserve dynamics remain underexplored. Hyperopic Reserve Deficiency: Age-stratified thresholds per national guidelines (14).

### 2.4 Statistical analysis

The SPSS27 software were used to statistically analyze the data. Statistical data were expressed as percentage (%), and inter-group comparisons were conducted by *χ*^2^ (chi-square) test or Fisher’s exact test. Using individual myopia events as the dependent variable, various baseline characteristics as covariates, and adjusting for age and gender where appropriate, a multiple logistic regression model was conducted to explore factors associated with myopia events. Multivariable logistic regression adjusted for covariates (*α* = 0.05, two-tailed). To investigate the predictive ability of myopia risk factors, receiver operating characteristic curves were plotted to calculate the area under the curve. A two-tailed *p* value less than 0.05 was considered statistically significant.

## 3 Result

### 3.1 Myopia prevalence

**Figure 2:**
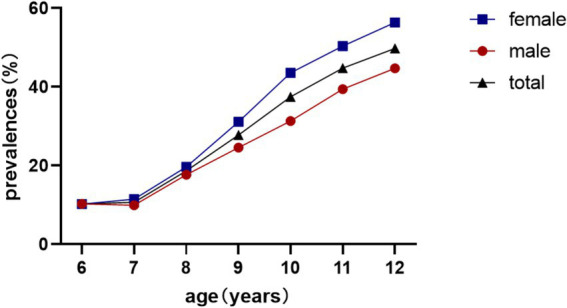
Prevalence of myopia in schoolchildren.

Overall myopia prevalence was 23.77%, escalating from 10.25% (age 6) to 49.77% (age 12) (*χ*^2^ = 1464.4, *p* < 0.001). Females exhibited higher rates (26.38% vs. 21.26%; *χ*^2^ = 54.3, *p* < 0.001; Table 1; Figure 2).

**Table 1:** Myopia prevalence.

Age	Male	Female	Total
	Myopia cases/male participants (%)	Myopia cases/female participants (%)	Myopia cases/total participants (%)
6	166/1613 (10.29)	162/1587 (10.21)	328/3200 (10.25)
7	142/1433 (9.91)	148/1278 (11.58)	290/2711 (10.70)
8	195/1103 (17.68)	222/1128 (19.68)	417/2231 (18.69)
9	302/1229 (24.57)	369/1186 (31.11)	671/2415 (27.78)
10	330/1054 (31.31)	446/1017 (43.85)	776/2071 (37.47)
11	442/1121 (39.43)	544/1080 (50.37)	986/2201 (44.80)
12	55/123 (44.72)	53/94 (56.38)	108/217 (49.77)
Total	1632/7676 (21.26)	1944/7370 (26.38)	3576/15046 (23.77)

### 3.2 Hyperopic reserve dynamics

**Figure 3:**
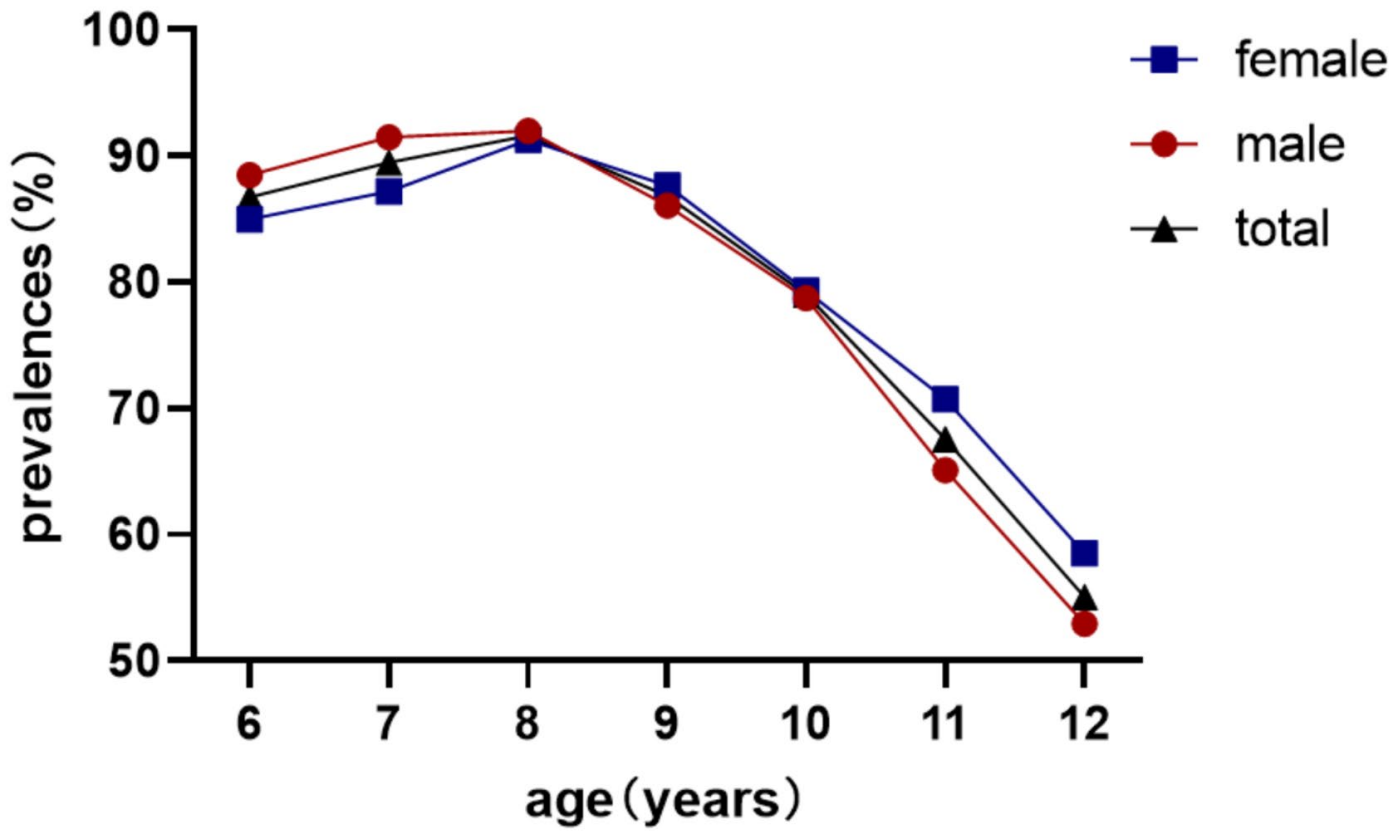
Prevalence of insufficient hyperopic reserve in non-myopic schoolchildren.

Reserve deficiency prevalence followed an inverted U-curve, peaking at 91.62% (age 8) before declining to 55.05% (age 12) (*χ*^2^ = 511.1, *p* < 0.001; Table 2). No sex difference was observed in reserve deficiency rates (85.08% vs. 84.68%; *χ*^2^ = 0.341, *p* = 0.559; Table 2; Figure 3).

**Table 2:** Detection rate of hyperopia reserve deficiency in non-myopic schoolchildren.

Age	Male	Female	Total
	Hyperopia reserve deficiency cases/male participants (%)	Hyperopia reserve deficiency cases/female participants (%)	Hyperopia reserve deficiency cases/total participants (%)
6	1280/1447 (88.46)	1211/1425 (84.98)	2491/2872 (86.73)
7	1181/1291 (91.48)	9,851,130 (87.17)	2166/2421 (89.47)
8	835/908 (91.96)	827/906 (91.28)	1662/1814 (91.62)
9	798/927 (86.08)	716/817 (87.64)	1514/1744 (86.81)
10	570/724 (78.73)	453/571 (79.33)	1023/1295 (79.00)
11	442/679 (65.10)	379/536 (70.71)	821/1215(67.57)
12	36/68 (52.94)	24/ 41 (58.54)	60/109 (55.05)
Total	5142/6044 (85.08)	4595/5426 (84.68)	9737/11470 (84.89)

### 3.3 Myopia conversion by reserve level

**Figure 4:**
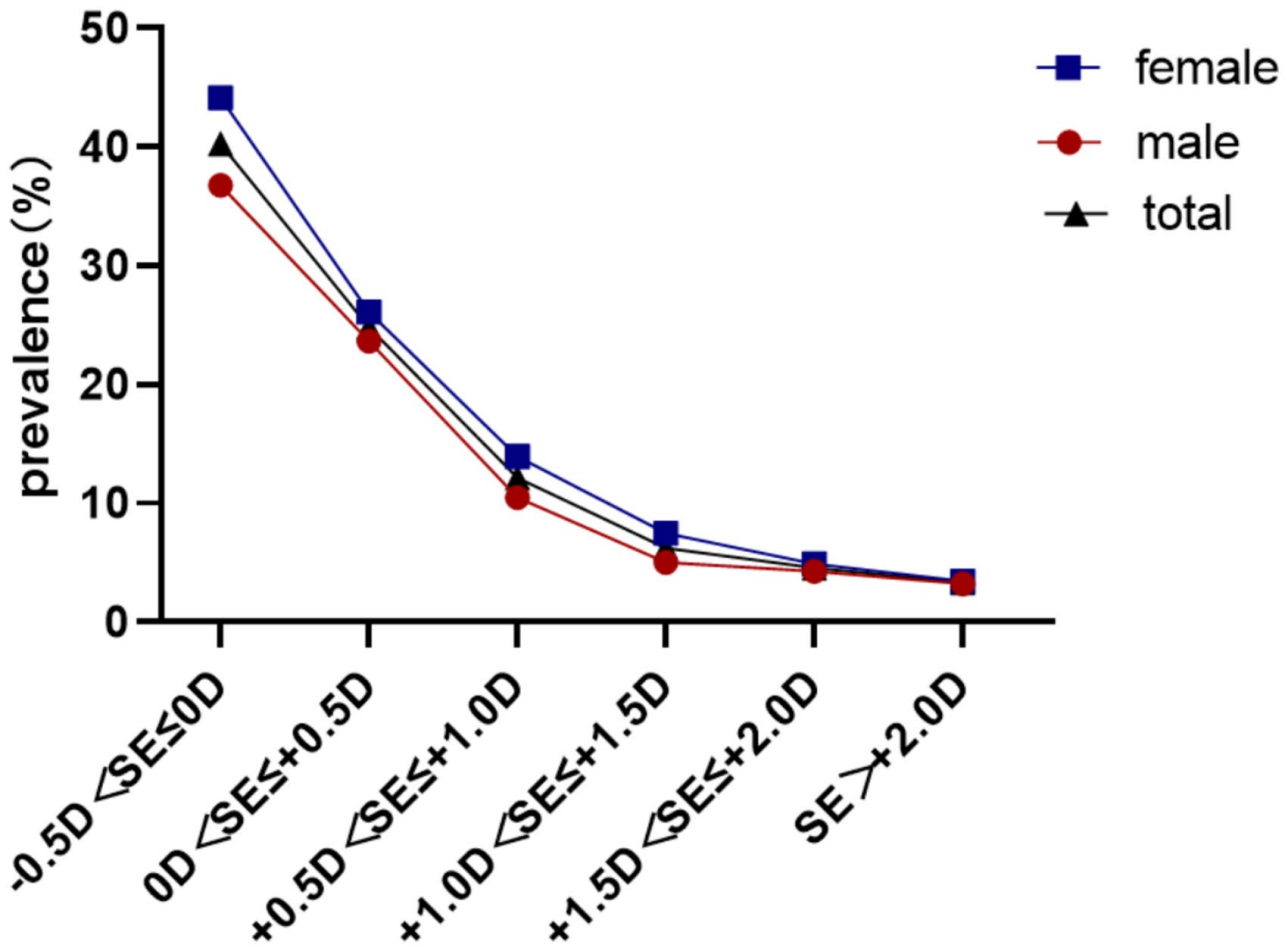
Two-year myopia incidence rates stratified by baseline hyperopic reserve levels.

Baseline reserve −0.5D to 0D predicted 40.27% myopia incidence, decreasing to 3.33% for reserve > + 2.00D (*χ*^2^ = 730.3, *p* < 0.001) Females demonstrated elevated conversion rates across all reserve strata (31.61% vs. 26.80%; *χ*^2^ = 32.0, *p*<0.001). As shown in Table 3, compared with the reference group (−0.5D < SE ≤ 0D, 40.27%), all groups with positive hyperopic reserve showed a significantly lower incidence of myopia over the two-year period (all*p* < 0.001). Moreover, a stronger baseline hyperopic reserve was associated with a lower incidence of myopia (Table 3; Figure 4).

**Table 3:** Rates of progression to myopia after 2 years with different reserves of hyperopia.

Diopter	Male	Female	Total	*p*
	*n* (%)	*n* (%)	*n* (%)	
−0.5D<SE ≤ 0D	1015/2760 (36.78)	1097/2485 (44.14)	2112/5245 (40.27)	Reference
0D<SE ≤ +0.5D	492/2082 (23.63)	479/1834 (26.12)	971/3916 (24.80)	<0.001
+0.5D<SE ≤ +1.0D	103/985 (10.46)	126/903 (13.95)	229/1888 (12.13)	<0.001
+1.0D<SE ≤ +1.5D	7/139 (5.04)	10/134 (7.46)	17/273 (6.23)	<0.001
+1.5D<SE ≤ +2.0D	2/47 (4.26)	2/41 (4.88)	4/88 (4.55)	<0.001
SE> + 2.0D	1/31 (3.23)	1/29 (3.45)	2/60 (3.33)	<0.001
Total	1620/6044 (26.80)	1715/5426 (31.61)	3335/11470 (29.08)	

*p*-values were calculated using the Chi-square test or Fisher’s exact test, as appropriate, for comparisons of the myopia incidence rate with the reference group (−0.5D < SE ≤ 0D).

### 3.4 Risk factor analysis

Parental myopia (maternal OR = 1.60, 95% CI:1.42–1.80, *p* < 0.001), paternal (OR = 1.35, 95% CI:1.20–1.54, *p* < 0.001), ≥10 h/day near work (OR = 1.49, 95% CI: 1.33–1.68, *p* < 0.001), ≥2 h/day screen time (OR = 1.17, 95% CI:1.04–1.30, *p* = 0.007), and high sugar intake (OR = 1.46, 95% CI:1.30–1.64, *p* < 0.001) significantly increased myopia risk. Protective factors included ≥2 h/day outdoor activity (OR = 0.47, 95% CI:0.40–0.55, *p* < 0.001), adequate sleep (OR = 0.84, 95% CI:0.75–0.95, *p* = 0.004) and whether doing ‘one punch, one foot, one inch’ when learning (OR = 0.63, 95% CI:0.56–0.71, *p*<0.001; Tables 4, 5).

**Table 4:** Univariate logistic regression analysis of myopia-related risk factors.

Risk Factors	Beta (Log odds coefficient)	OR (95% CI)	*p*
Maternal myopia			
No	Reference	Reference	
Yes	0.56	1.74 (1.55–1.95)	<0.001
Paternal			
No	Reference	Reference	
Yes	0.42	1.53 (1.35–1.72)	<0.001
Daily learning time			
<10 h	Reference	Reference	
≥10 h	0.47	1.60 (1.43–1.79)	<0.001
Daily exercise time			
<2 h	Reference	Reference	
≥2 h	−0.96	0.39 (0.33–0.45)	<0.001
Daily screen time			
<2 h	Reference	Reference	
≥2 h	0.18	1.20 (1.08–1.33)	0.01
Whether like sugar			
No	Reference	Reference	
Yes	0.47	1.60 (1.42–1.79)	<0.001
Daily sleep time			
≤8 h	Reference	Reference	
>8 h	−0.26	0.77 (0.69–0.86)	<0.001
Whether doing ‘one punch, one foot, one inch’			
No	Reference	Reference	
Yes	−0.63	0.53 (0.48–0.60)	<0.001

**Table 5:** Multivariable logistic regression analysis of myopia-related risk factors.

Risk Factors	Beta	OR (95% CI)	*P*
Maternal myopia			
No	Reference	Reference	
Yes	0.47	1.60 (1.42–1.80)	<0.001
Paternal			
No	Reference	Reference	
Yes	0.3	1.35 (1.20–1.54)	<0.001
Daily learning time			
<10 h	Reference	Reference	
≥10 h	0.4	1.49 (1.33–1.68)	<0.001
Daily exercise time			
<2 h	Reference	Reference	
≥2 h	−0.76	0.47 (0.40–0.55)	<0.001
Daily screen time			
<2 h	Reference	Reference	
≥2 h	0.15	1.17 (1.04–1.30)	0.007
Whether like sugar			
No	Reference	Reference	
Yes	0.38	1.46 (1.30–1.64)	<0.001
Daily sleep time			
≤8 h	Reference	Reference	
>8 h	−0.17	0.84 (0.75–0.95)	0.004
Whether doing ‘one punch, one foot, one inch’			
No	Reference	Reference	<0.001
Yes	−0.46	0.63 (0.56–0.71)	

## 4 Discussion

Ocular biometric characteristics, such as axial length (AL), corneal curvature, and lens power, are among the most important factors affecting the refractive state of the eye. During infancy and early childhood, most children have a physiological hyperopia of approximately +2.00 diopters (D), which is defined as hyperopic reserve. Balancing changes in AL and ocular refractive components (including the cornea and lens) leads to emmetropia. As children age, the refractive power of the eye gradually increases, and the hyperopic reserve gradually decreases. During neonatal development, due to the shortening of the axial length (averaging 16.8 millimeters at birth and 23.6 millimeters in adulthood) and the immaturity of the corneal curvature, the human ocular system exhibits physiological hyperopia (15). This hyperopic reserve undergoes systematic depletion through coordinated ocular growth processes - principally axial elongation and corneal flattening - achieving emmetropization typically by age 12 through homeostatic visual feedback mechanisms (15). Premature exhaustion of this refractive buffer (≤ + 0.50D by age 6) precipitates myopic progression via dysregulated scleral remodeling. Contemporary evidence positions hyperopic reserve as a critical biomechanical safeguard against pathological ocular expansion, demonstrating strong predictive validity for myopia onset when depleted below age-specific thresholds (7).

This study revealed an age-dependent increase in myopia prevalence among primary school students (aged 6–12 years) in Xi’an during 2021, with female students demonstrating significantly higher rates compared to their male counterparts, consistent with previous epidemiological reports (15). This discrepancy may be attributed to gender-specific behavioral patterns: female students tend to engage more frequently in near-work activities (e.g., reading and drawing) while males predominantly participate in outdoor sports (e.g., soccer, basketball, and badminton). Extended outdoor exposure enhances light-induced dopamine release, which has been mechanistically linked to myopia suppression (16–20). Our multivariate logistic regression analysis quantitatively confirmed that prolonged outdoor activities significantly reduced myopia risk. Furthermore, the observed gender differences may be exacerbated by female students’ consumption of high-sugar foods, such as bubble tea and chocolate. Multivariate analysis identified this as an independent risk factor for the development of myopia, which is consistent with existing evidence of a positive correlation between dietary sugar intake and myopia progression (21). A previous study in French school children showed an increased probability of myopia in girls and an unexpected decrease in the probability of myopia in boys. This may be because the frequency of carbohydrate intake does not truly reflect chronic hyperglycemia in boys, who are more active than girls at all ages (22).

The longitudinal prevalence pattern illustrated in Figure 4 demonstrates a gradual increase in myopia detection rates from ages 6–7 years, followed by marked acceleration from age 7–8 years onward. These epidemiological observations strongly suggest the critical importance of initiatory refractive status monitoring and myopia prevention strategies during the early school years (6–7 years old), which could effectively mitigate subsequent high-grade myopia development in later academic stages.

This study identified an age-dependent developmental trajectory of insufficient hyperopic reserve among non-myopic children aged 6–12 years, characterized by a progressive increase in prevalence from ages 6–8 years (peaking at approximately 8 years), followed by a subsequent decline, consistent with established physiological patterns of ocular development (23, 24). Notably, gender-specific variations emerged across different age cohorts: female students exhibited lower prevalence rates compared to males in the younger cohort (6–8 years), whereas this pattern reversed in older children (9–12 years) with females demonstrating higher rates. These findings are consistent with previous epidemiological observations by Wang (23), who observed a negative correlation between a decrease in the prevalence of hyperopia reserve and an increase in myopia rate in the older children. This may reflect a natural progression pattern, where a significant portion of children with depleted hyperopia reserve have transitioned to clinical myopia. This biological transformation process is consistent with the current understanding of the development of refractive errors during eye maturation.

Our longitudinal analysis demonstrated a strong association between baseline hyperopic reserve and incident myopia in non-myopic schoolchildren, indicating a dose-dependent relationship between reduced hyperopic reserves and increased myopia risk. These findings align with the 5-year cohort study by Li (13), which reported progression rates of 4.6% versus 94.3% for equivalent SE categories in first-grade students (*N* = 2,628), confirming the accelerated myopiagenic progression in low-reserve populations.

Notably, female students consistently demonstrated higher overall myopia incidence across all reserve categories compared to males (*p* < 0.05), a gender disparity corroborated by Li (25). This persistent pattern highlights the necessity for gender-specific preventive strategies focusing on hyperopic reserve preservation in female students.

Current evidence-based strategies to preserve hyperopic reserves and delay premature myopia onset primarily involve three key interventions: enhanced outdoor activity engagement (≥2 h/day) (16–20), restricted near-work duration (26, 27), and low-concentration atropine therapy (0.01–0.05%) (28–31). Our multivariate logistic regression models identified prolonged near-work exposure as an independent risk factor for myopia progression, necessitating coordinated efforts across educational institutions, families, and students. A recent study on Chinese school-aged children has shown that increasing outdoor time can effectively reduce the onset and progression of myopia in children with myopia (32). Therefore, schools should prioritize recess and enforce outdoor activities during the 10-min break and the extended 30-min break, rather than implementing excessive indoor restrictions due to incorrect safety concerns - such practices can actually increase risks to eye health.

Parental supervision should ensure adherence to the 20–20-20 rule (20-s visual breaks every 20 min of near-work) and limit recreational screen time (OR = 1.17, 95% CI:1.04–1.30 for ≥2 h/day). Academic activities must maintain proper reading posture (30–40 cm viewing distance) as our analysis confirmed the protective effect of ergonomic practices (OR = 0.63, 95% CI:0.56–0.71) and adequate sleep duration (OR = 0.84, 95% CI:0.75–0.95).

These findings carry significant clinical and public health implications. Our results suggested that regularly monitoring to detect myopia, combined with interventions to increase outdoor time, can serve as an effective strategy for myopia prevention. A tiered prevention framework requires medical institutional participation through:

1. Establishment of longitudinal refractive profiles for monitoring hyperopic reserve dynamics.
2. Personalized myopia control protocols for subclinical cases.
3. School-based ophthalmology outreach programs delivering science-backed prevention guidelines.

This tripartite collaboration model (school-family-hospital) ensures systematic implementation of optical biometric monitoring, behavioral modification, and pharmacological intervention when needed.

This study possesses notable strengths including a substantial sample size (*N* = 15,046) that enhances the generalizability of our findings to school-aged populations. However, several methodological limitations warrant consideration. First, the utilization of non-cycloplegic autorefraction for large-scale screening, while operationally efficient, may systematically underestimate refractive hyperopia compared to gold-standard cycloplegic measurements (33, 34), potentially leading to overestimation of hyperopic reserve depletion rates. Second, the 2-year observational period restricts our ability to characterize long-term myopia progression patterns. We intend to address this through extended follow-up to determine cumulative incidence rates across hyperopic reserve subgroups.

In summary, our epidemiological survey reveals two major public health challenges faced by children in Xi’an: an alarmingly high prevalence of myopia among children aged 6–12, insufficient hyperopic reserve, and a high conversion rate of myopia within 2 years. These findings collectively indicate the necessity for paradigm-shifting prevention strategies, such as early optical monitoring and enhanced screening for high-risk schoolchildren, as well as personalized interventions. Through our analysis, this tiered prevention framework, integrating school screening, community ophthalmology networks, and family education initiatives, may reduce myopia events in the primary school cohort.

## Data Availability

The original contributions presented in the study are included in the article/supplementary material, further inquiries can be directed to the corresponding author/s.
